# The single-nucleotide polymorphism (GPX4c718t) in the glutathione peroxidase 4 gene influences endothelial cell function: Interaction with selenium and fatty acids

**DOI:** 10.1002/mnfr.201300216

**Published:** 2013-08-12

**Authors:** Lynne K Crosley, Shabina Bashir, Fergus Nicol, John R Arthur, John E Hesketh, Alan A Sneddon

**Affiliations:** 1 Micronutrients Group, Lifelong Health Division, Rowett Institute of Nutrition and Health, University of AberdeenAberdeen, UK; 2 Institute for Cell and Molecular Biosciences and Human Nutrition Research Centre, The Medical School, Newcastle University, Tyne and WearUK

**Keywords:** Endothelial, Glutathione peroxidase 4, Monocyte, Selenium, Single nucleotide polymorphism

## Abstract

**Scope:**

Selenium (Se) is incorporated into selenoproteins as selenocysteine, which requires structures in the 3′-untranslated region (3′-UTR) of selenoprotein mRNAs. The functional consequences of a single nucleotide polymorphism (SNP) within the 3′-UTR of the selenoprotein *GPX4* gene (GPX4c718t) was assessed in human umbilical vein endothelial cells (HUVECs) and monocytes from human volunteers.

**Methods and results:**

HUVEC and monocytes homozygous for the T- or C-variant of the GPX4c718t SNP were assessed for monocyte–endothelial cell adhesion, expression of VCAM-1 and sensitivity to oxidative challenge. Interaction of the SNP with Se and different PUFA and effects on selenoprotein expression were also investigated. HUVEC and monocytes homozygous for the T-variant showed elevated adhesion levels compared to cells of the C-variant. This effect was modified by Se and PUFA. HUVEC homozygous for the T-variant showed elevated levels of VCAM-1 protein in the presence of arachidonic acid, were more sensitive to oxidative challenge and showed Se-dependant changes in lipid peroxide levels and expression of additional selenoproteins.

**Conclusion:**

These findings demonstrate functional effects of the GPX4c718t SNP in endothelial cells and may suggest that individuals with the TT genotype have impaired endothelial function and are at greater risk of vascular disease compared to individuals with the CC genotype.

## 1 Introduction

Inappropriate diet may account for a significant proportion of cardiovascular disease (CVD) [1], therefore understanding how specific nutrients or dietary regimes modulate cardiovascular function is an important area. Genetic make-up also contributes to CVD risk and the interaction of functional genetic variants with dietary components is likely to underlie many multifactorial diseases including CVD [2].

Suboptimal intake of the micronutrient selenium (Se) has been associated with greater mortality as well as increased risk of diseases including CVD, prostate, and colorectal cancers [3–5]. The metabolic functions of Se are thought to be due to its presence as selenocysteine (Sec) in selenoproteins of which there are 25 in humans. Most selenoproteins, including glutathione peroxidases (GPX) and thioredoxin reductases (TR), have antioxidant and redox functions that protect cells from oxidative stress and cell damaging agents [6]. Consequently, since low dietary intakes of Se may lower antioxidant status this may increase the risk of diseases associated with oxidative damage, namely, CVD and cancer.

Se is incorporated into selenoproteins as the amino acid Sec during protein synthesis. Sec is encoded by a UGA codon, which requires a specific RNA structure in the 3′-untranslated region (3′-UTR) of selenoprotein mRNAs termed a Sec insertion sequence. Under Se-limiting conditions, Se utilization is specifically prioritized between and within tissues resulting in preferential expression of some selenoproteins to the detriment of others [7]. This phenomenon is referred to as the selenoprotein hierarchy and is in part due to differences in the interaction of the different selenoprotein 3′-UTR sequences with the Sec incorporation machinery [6–8]. Accordingly, single nucleotide polymorphisms (SNPs) within such a 3′-UTR may have functional effects through not only affecting synthesis of the selenoprotein encoded by that mRNA but also by affecting the synthesis of additional selenoproteins because of effects on the hierarchy [8,9].

GPX4 (phospholipid hydroperoxide glutathione peroxidase) is an intracellular antioxidant selenoprotein that can reduce lipid hydroperoxides within cell membranes, [reviewed in [10]). GPX4 can also potentially modulate inflammation through regulating leukotriene biosynthesis and cytokine-signaling pathways [10,11]. A T/C variation (*rs713041*) in the region of the GPX4 gene that corresponds to the 3′-UTR of the mRNA is present in Asian and Caucasian populations [11,12]. Evidence suggests that this SNP is functional because studies have shown it to be associated with differences in the levels of 5-lipoxygenase metabolites in lymphocytes [11] and differences in driving selenoprotein synthesis in reporter gene studies [13,14]. Moreover, results from a supplementation study suggest that the SNP can affect expression of GPX4 and GPX1 in human lymphocytes, suggesting that the variants differ in their ability to interact with the Se incorporation machinery [9]. Further studies have also provided evidence to correlate this SNP with disease; the SNP is associated with susceptibility to colorectal cancer and to increased risk of death in breast cancer patients [13,15]. More recently, the SNP was also shown to be a predictor of stroke in patients with hypertension [16].

The aim of the present work was to investigate the potential role of the GPX4c718t SNP in cardiovascular function by determining whether this SNP has functional effects in human vascular endothelial cells (ECs) and to examine how these are modified by different dietary components including Se and fatty acids; fatty acids are known to modulate monocyte adhesion to endothelial cells [17,18] and they can also regulate GPX4 expression [19]. The recruitment of monocytes from the circulation and their binding to and transmigration across the lining of EC covering the artery into the intimal space of the arterial wall is a key early event in atherosclerosis development [20]. In order to determine the effect of the GPX4c718t SNP on this process, Human umbilical vein endothelial cells (HUVECs) genotyped for GPX4c718t and homologous for either T (TT) or C (CC) were assayed for their ability to bind monocyte cells under both basal and activated conditions.

## 2 Materials and methods

### 2.1 Cell culture

Both pooled (from five different donors) and single-donor primary HUVEC (Greiner Bio-one) were routinely cultured as per the manufacturer's protocol in EGM-2 bulletkit medium (which contains Se). For Se deficient experiments, HUVEC cultured in EGM-2 medium were first washed three times in 1× PBS (Sigma-Aldrich) and then grown in M199 medium (Gibco) (which contains no Se) with 10% fetal calf serum (FCS) added (Sigma-Aldrich). FCS itself contained Se at 175 nM but this Se form was shown not to be bio-available in that it did not support GPX activity. However, GPX1 activity was maximally stimulated after the addition of 40 nM sodium selenite (data not shown). U937 cells (Sigma-Aldrich) were maintained in RPMI-1640 medium (Sigma-Aldrich) with 10% FCS, penicillin (1 U/mL) and streptomycin (1 μg/mL; Gibco).

### 2.2 Recruitment and blood sampling of human volunteers

Twenty healthy nonsmoking male volunteers aged between 25 and 55 years old were recruited from the general population in the Aberdeen area. Exclusion criteria included those taking aspirin or other anti-inflammatory drugs or medicines known to alter the immune/haemostatic function; excessive alcohol consumption (>30 units/wk); and those taking fatty acid, multivitamin or other herbal supplements within the previous 6 wk. Volunteers were asked to attend the Human Nutrition Unit at the Rowett Institute of Nutrition and Health on two separate occasions. At the first visit, a mouth swab was taken for GPX4c718t genotyping of buccal cell DNA. At the second visit, a fasted peripheral blood sample (40 mL) was drawn from the antecubital vein into EDTA-tubes (BD Vacutainer; Becton-Dickinson) and processed for monocyte isolation and adhesion assays. Written informed consent was obtained from all participants and the study was double-blinded. The study protocol was approved by the North of Scotland Research Ethics Committee (NOSRES; REC reference 08/0801/162).

### 2.3 Monocyte isolation from volunteers

Monocytes were isolated from fasted blood samples using OptiPrep™ as per the manufacturer's protocol (Axis-Shield). Flow cytometry using an anti-CD14-FITC antibody (BD Biosciences) routinely demonstrated that >85% of the cells were CD14^+^.

### 2.4 DNA extraction and genotyping

Buccal cell DNA was isolated from volunteer mouth swabs using QIAmp (Qiagen) according to the manufacturer's specifications. Single donor HUVEC and U937 DNA were isolated from around 5 × 10^5^ cells using the QIAprep DNA kit (Qiagen) and protocol. To determine the genotype for GPX4 718, a PCR was performed with 100 ng template genomic DNA, using forward (5′-GACCTGCCCCACTATTTCTA-3′) and reverse (5′-GTCTGTTTATTCCCACAAGG-3′) primers and an Expand High-Fidelity PCR system (Roche) in a Thermo-Hybaid Px2 thermocycler under the following conditions: an initial denaturating step at 94°C for 4 min, followed by 30 cycles of denaturation (94°C for 30 s), annealing (53.5°C for 30 s), and extension (72°C for 1 min). The PCR was completed by a final extension cycle at 72°C for 7 min. An aliquot of each PCR product was subjected to electrophoresis in a 1× Tris-acetate-EDTA 1.5% agarose gel and visualized with ethidium bromide as a 221-bp fragment. PCR products were purified using a Qiagen PCR purification kit as per the manufacturer's protocol prior to DNA sequencing (Beckman Coulter CEQ 8000) to confirm the GPX4c718t genotype. Of the 20 human volunteers recruited onto the study, three were CC and five were TT for the GPX4c718t SNP. Out of 18 aliquots of single donor HUVECs analyzed, five were CC and five were TT for the GPX4c718t SNP.

### 2.5 Selenium deficiency and repletion of genotyped HUVEC and selenoprotein assay

HUVECs genotyped for GPX4c718t (CC or TT) were grown to subconfluence in EGM-2 medium containing bulletkit components and then subcultured into Se-deficient conditions (M199 medium containing 10% FCS) (d0). Cells were kept in this medium (fresh media replenished every 2 days) for up to 9 days. After 5 days, 40 nM Se (as sodium selenite) was added to some flasks for further 4 days (d9). Cells in both flasks were harvested at day 0, 5, 7, and 9 and cell pellets were assayed for total protein and selenoenzyme levels. HUVEC extracts were assayed for GPX1 activity and GPX4 and TR1 protein by a competitive ELISA as described previously [14]. Total protein content was measured by the bicinchoninic acid assay method (Thermo Scientific).

### 2.6 Fatty acid treatments and monocyte-endothelial adhesion assays

Pooled HUVECs were used to perform the monocyte–endothelial adhesion assay when using genotyped monocytes from the human volunteers. For the assay using the genotyped HUVECs, U937 monocytes were used to test for adhesion. HUVECs were grown in Se-deficient media with or without Se (40 nM) for 5 days. After 3 days, cells were either treated with 10 μM arachidonic acid (ARA) (22:4*n*-6), linoleic acid (LA) (18:2*n*-6), docosahexaenoic acid (DHA) (22:6*n*-3), or ethanol alone for 2 days. Fatty acids (Cayman Chemicals; 99% pure free) were dissolved in 100% ethanol and stored at −20°C under nitrogen in the dark. Cells were then plated overnight to confluence into 96-well plates and some wells were challenged with TNF-α (Source Biosciences, 5 ng/mL) for 6 h and then all wells were washed twice in prewarmed M199 medium and then 3 × 10^4^ labeled monocytes/well were added for 30 min. Monocytes were previously labeled with 5 μM BCECF-AM (Invitrogen) for 30 min, and then washed three times with 1× PBS containing 1% FCS before resuspending in M199 medium. After incubation of the HUVEC with the monocytes, wells were washed three times in 1× PBS containing 1% FCS to remove unbound monocytes, then fluorescence was measured (excitation/emission was 488/530 nm). Control wells containing either no HUVECs or no monocyte additions were used to calculate background fluorescence.

### 2.7 Cell lipid peroxidation measurement

HUVECs genotyped for GPX4c718t were cultured in M199 medium plus 10% FCS in the presence or absence of 40 nM sodium selenite for 5 days. Cells were seeded onto 96-well plates at 3 × 10^4^ cells/cm^2^ in the same medium and left overnight. Cells were then incubated in the presence of 5 μM C11-BODIPY^581/591^ (4,4-difluoro-5-(4-phenyl-1,3-butadienyl)-4-bora-3a,4adiaza-s-indacene-3-undecanoic acid) (Invitrogen/Molecular Probes) for 30 mins at 37°C. After rinsing cells twice in enriched DPBS^+^ (137 mM NaCl, 2.7 mM KCl, 0.5 mM MgCl_2_, 0.9 mM CaCl_2_, 1.5 mM KH_2_PO_4_, 8.1 mM Na_2_HPO_4_, and 5 mM glucose, pH 7.4), oxidation of C11-BODIPY^581/591^ was determined by measuring fluorescence at 590/520 nm (excitation/emission). Some cells were also challenged with 0.1 mM *tert*-butylhydroperoxide (TBH) (Sigma-Aldrich) in DPBS^+^ to induce oxidative stress. Wells containing cells only were used to calculate background fluorescence levels.

### 2.8 Cell viability assays

A total of 96-well plates were seeded with 6 × 10^4^ cells/cm^2^ of genotyped HUVECs and grown in M199 medium plus 10% FCS in the presence or absence of 40 nM sodium selenite. After 5 days, cells were challenged with 0.3 mM TBH for 4 h and then fresh medium was added to the cells. Cell viability was assessed by the addition of 20 μL of CellTiter-Blue® reagent (Promega) for 4 h and absorbance was measured at 560 nm. This assay is based on the ability of living cells to convert resazurin, a redox dye, into a fluorescent end product (resorufin). Nonviable cells rapidly lose metabolic capacity and do not generate a fluorescent signal.

### 2.9 Protein expression analysis

Protein extraction from HUVEC and Western blot analysis was done as described previously [18] using 10% SDS-PAGE and a rabbit anti-VCAM1 antibody (Insight Biotechnology) and mouse anti-β-Actin antibody (Sigma-Aldrich) as a loading control.

### 2.10 Statistical analysis

Experiments were carried out at least three times in duplicate or triplicate and the results are presented as means ± SEM. Comparison of several treatments was evaluated by analysis of variance (ANOVA) on the log-transformed data, followed by post-hoc *t*-tests.

## 3 Results

### 3.1 Effect of GPX4c718t genotype in HUVEC on adhesion to monocytes: Effect of Se and PUFA

**Figure 1:**
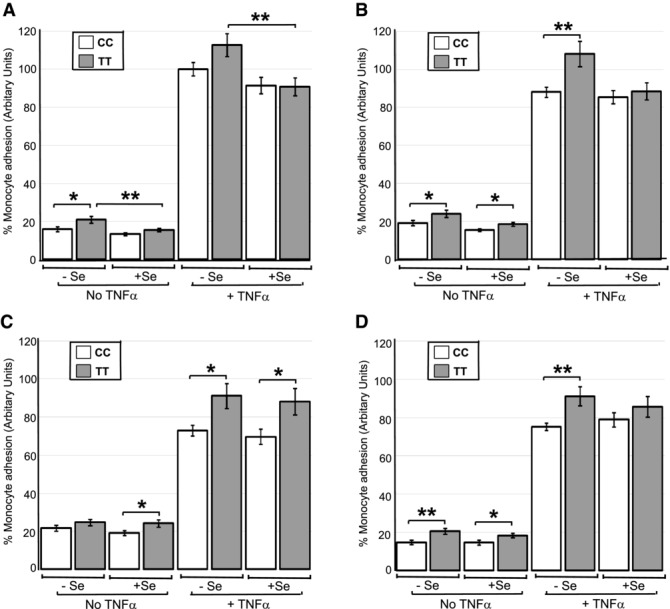
Effect of the GPX4c718t SNP in HUVEC and fatty acid pretreatment on monocyte adhesion. HUVECs genotyped for GPX4c718t (CC or TT) were grown to confluence under Se replete or Se deficient conditions (+Se and −Se) and challenged with TNF-α and the number of adhering monocytes was assayed. Cells were either pretreated with no fatty acid (A) or had prior pretreatment with linoleic acid (B), ARA (C), or docosahexanoic acid (D) at 10 μM for 48 h. All values are expressed as a percentage (± SEM) of the value obtained for the level of monocyte adhesion to cells of the CC genotype after TNF-α challenge in the absence of Se and fatty acid. Statistical analysis was carried out by four-way ANOVA on the log-transformed data followed by post hoc *t*-tests (*p < 0.05, and **p < 0.01).

The U937 monocytic cell line was used in these experiments and genotype analysis of these cells established it to have the CC genotype at the GPX4c718t SNP. HUVEC genotyped for GPX4c718t were assayed for their ability to bind U937 monocytes with and without a TNF-α challenge. This latter challenge mimics the inflammatory conditions occurring within the endothelium resulting in increased monocyte adhesion that contributes to development of CVD. We were also interested in determining whether PUFA-induced changes in monocyte–endothelial adhesion were modified by the GPX4c718t SNP. PUFA such as DHA, LA, and arachidonic acid (ARA) are known to modulate monocyte–endothelial adhesion [17,18,30,31], a process which may be mediated through the PUFA-induced formation of distinct eicosanoid species [21]. Since GPX4 activity has also been shown to modulate cellular eicosanoid levels [11], this provided a rational for investigating the interaction between the SNP and PUFA on the adhesion process. Four-way ANOVA analysis demonstrated that HUVEC with the TT genotype exhibited an overall 15% increase in adhesion (*p* < 0.001) compared to the CC genotype (Fig. 1). Additionally, there was an effect of fatty acid, Se, and TNF-α on adhesion levels and an interaction of fatty acid and TNF-α (all *p* < 0.001) and genotype with TNF-α (*p* < 0.05) with all other interactions being nonsignificant.

In Se-deficient cells under basal conditions, monocyte adhesion to HUVECs with the TT genotype was significantly greater (32%; *p* < 0.05) compared with cells with the CC genotype (Fig. 1A). Under Se-replete conditions, there was no significant difference in monocyte adhesion between cells of either genotype. However, in the presence of Se, cells with the TT genotype exhibited a 26% decrease (*p* < 0.01) in monocyte adhesion compared to TT cells in the absence of Se (Fig. 1A). This latter effect was also manifest in cells challenged with TNF-α; cells of the TT genotype exhibited lower monocyte adhesion (24%; *p* < 0.01) after Se supplementation compared with the absence of Se (Fig. 1A). LA pretreatment of the HUVEC had no additional effects on the level of adhesion compared to treatment without fatty acid (Fig. 1B). ARA pretreatment invoked an average 37% increase in adhesion in the absence of TNF-α challenge irrespective of Se presence (*p* < 0.05 or less), whereas it invoked decreased monocyte adhesion (average 18%) in the presence of TNF-α (*p* < 0.01 or less) except in Se-replete, TT cells (Fig. 1C). With DHA pretreatment, there were no differences in adhesion levels in the absence of TNF-α challenge compared with no fatty acid pretreatment, whereas upon TNF-α challenge, HUVECs of either genotype exhibited a reduction in adhesion (*p* < 0.05) in the absence but not in the presence of Se (Fig. 1D).

### 3.2 Effect of GPX4c718t genotype in HUVEC on VCAM-1 protein expression

**Figure 2:**
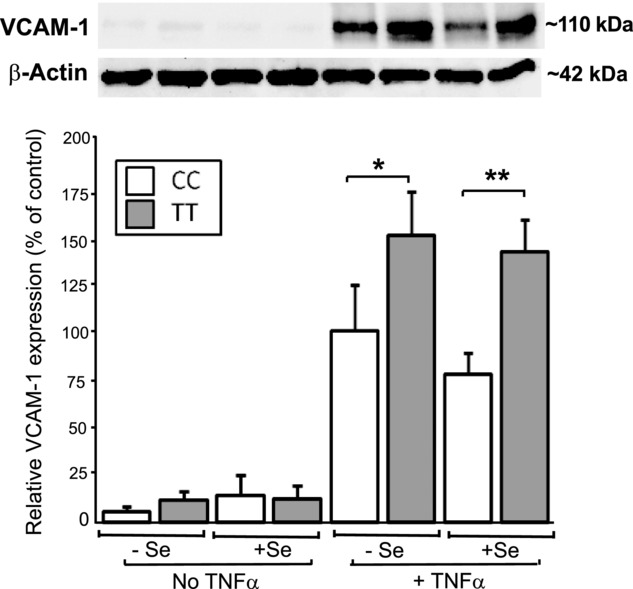
Effect of the GPX4c718t SNP in HUVEC on VCAM-1 protein levels after ARA pretreatment. HUVECs genotyped for GPX4c718t (CC or TT) were grown to confluence under Se replete or Se-deficient conditions (+Se and −Se) and were pretreated with ARA at 10 μM for 48 h. After challenge with TNF-α, the levels of VCAM-1 protein were assessed with representative Western blots shown above. All values are expressed as a percentage (± SEM) of the value obtained for VCAM-1 levels in HUVEC with the CC genotype after TNF-α challenge in the absence of Se. Statistical analysis was carried out by three-way ANOVA followed by post hoc *t*-tests (*p < 0.05 and **p < 0.01).

To gain insight into the mechanism involved in the effect of the GPX4c718t SNP in HUVEC on monocyte adhesion, the protein levels of VCAM-1 were assayed in cells pretreated with ARA as this treatment showed the greatest genotype difference on adhesion levels (Fig. 1). Three-way ANOVA analysis showed HUVEC with the TT genotype exhibited increased VCAM-1 levels overall compared to those with the CC genotype (*p* < 0.006). Additionally, there was an effect of TNF-α (*p* < 0.001) and an interaction of genotype and TNF-α (*p* < 0.009), with TT cells showing increased expression of VCAM-1 in the presence of TNF-α (Fig. 2).

### 3.3 Effect of monocyte GPX4c718t genotype on adhesion to EC

**Figure 3:**
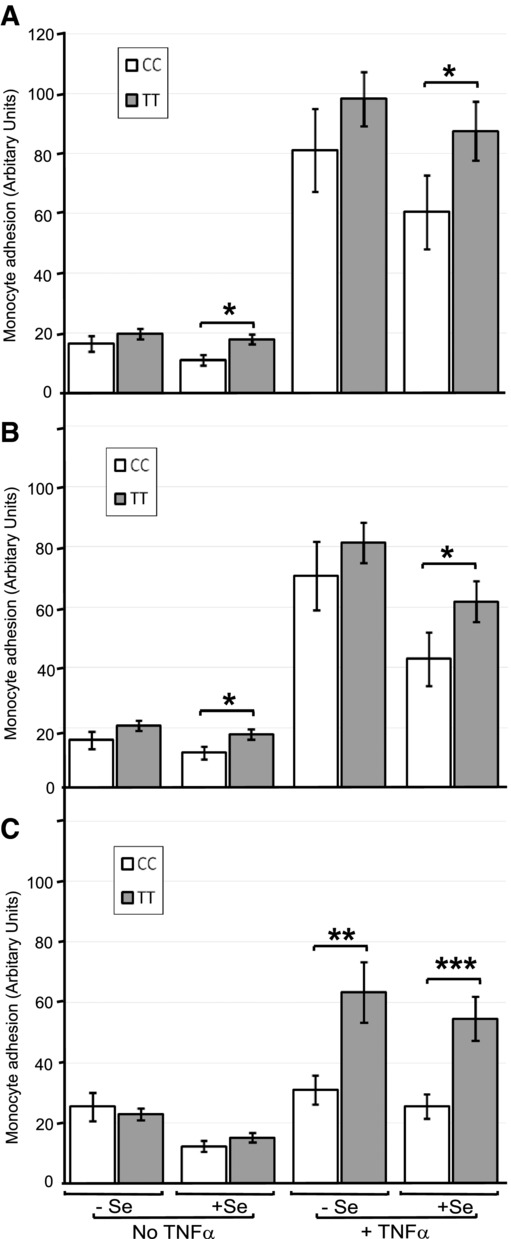
Effect of the GPX4c718t SNP in monocytes and EC fatty acid pretreatment on adhesion to HUVECs. Monocytes from volunteers genotyped for GPX4c718t (CC or TT) were isolated and added to HUVECs grown in the presence or absence of Se (+Se and −Se) and challenged with TNF-α and the number of adhering monocytes was assayed. Cells were either pretreated with no fatty acid (A) or pretreated with either linoleic acid (B) or ARA (C) at 10 μM for 48 h. Values are expressed as means ± SEM. Statistical analysis was carried out by four-way ANOVA on the log-transformed data followed by post hoc *t*-tests (*p < 0.05, **p < 0.01 and ***p < 0.001).

The effect of the GPX4c718t SNP on monocyte function was assessed by assaying the adhesion of monocytes isolated from genotyped human volunteers to HUVECs (isolated from five nongenotyped donors). Again, HUVECs were either pretreated with LA or ARA or untreated to investigate the response to fatty acids. Four-way ANOVA analysis demonstrated that TT monocytes exhibited an overall 39% increase in adhesion compared to CC monocytes (*p* < 0.001). Additionally, there was an effect of fatty acid, Se, and TNF-α on adhesion levels and an interaction of fatty acid and TNF-α (all *p* < 0.001) with all other interactions being nonsignificant. Without fatty acid pretreatment of the HUVEC, monocytes with the CC genotype showed decreased adhesion to HUVEC in the presence of Se, irrespective of TNF-α challenge (*p* < 0.05; Fig. 3A). LA pretreatment of HUVEC was without additional effect when compared to treatment without fatty acid (Fig. 3A versus 3B). However, pretreatment of HUVEC with ARA, while having no effect on adhesion levels in the absence of TNF-α, had a significant lowering effect on adhesion levels in the presence of TNF-α (all *p* < 0.05 or greater) which was more prevalent in cells of the CC genotype compared with those of the TT genotype (an average reduction of 60% versus 37% respectively; Fig. 3C).

### 3.4 Effect of GPX4c718t SNP on GPX4, GPX1, and TR1 selenoenzyme levels

**Figure 4:**
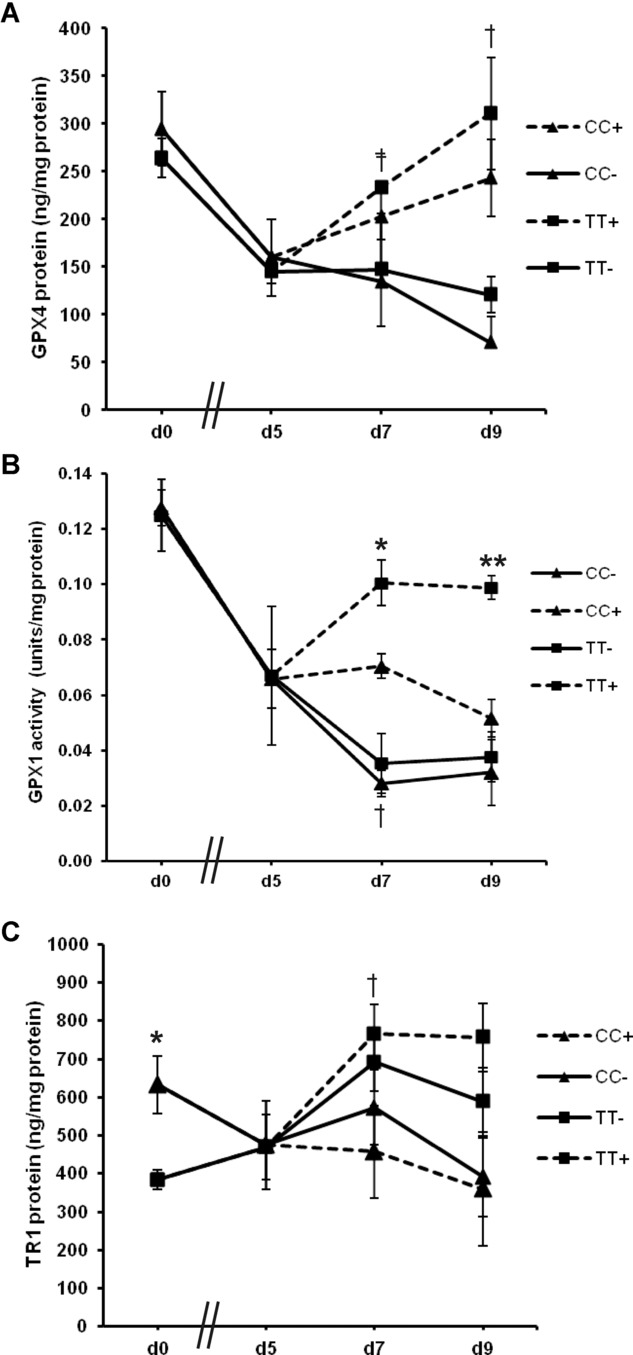
Effect of GPX4 718 SNP on GPX4, GPX1, and TR1 selenoenzyme levels. Genotyped HUVECs (CC or TT at GPX4 718) were initially cultured in Se replete medium (d0). Cells were then washed and cultured in Se deficient medium for 5, 7, or 9 days (d5, d7, d9). After five days of deficiency, some cells were supplemented with 40 nM Se for two days (d7+) or four days (d9+). At each time point, the levels of GPX4 protein (A), GPX1 activity (B), and TR1 protein (C) were measured. Values shown are means ± SEM. Student's *t*-test was used to test for significant differences (*p < 0.05 and **p < 0.01 for comparison of CC and TT genotypes and ^†^*p* < 0.05 and ^‡^*p* < 0.01 when compared to value at d5).

To establish whether the GPX4c718t SNP affected GPX4 levels in ECs, HUVEC homozygous for CC or TT at position 718 were grown in the presence and absence of Se and GPX4 protein levels determined by ELISA. In addition, cells made Se deficient for 5 days were then repleted with Se for further 4 days in order to assess the response of GPX4 levels to Se repletion. There were no differences in GPX4 protein in cells with the CC genotype compared with cells with the TT genotype in either Se adequate conditions or in those deficient for Se after 5 days (Fig. 4A, d0, and d5). However, in cells of both genotypes, GPX4 protein was downregulated by 45% (*p* < 0.05) after Se deficiency for 5 days (Fig. 4A). Further deficiency for 7 or 9 days did not result in any further statistically significant changes in GPX4 levels in either genotype (Fig. 4A). GPX4 levels increased significantly 2 days (d7) (*p* < 0.01) and 4 days (d9) (*p* < 0.05) after Se repletion in HUVEC with the CC genotype compared with levels at d5 but no significant increase was found in the cells of TT genotype (Fig. 4A). However, there were no significant differences in GPX4 protein levels between genotypes after Se repletion. Since there is a hierarchy in selenoprotein synthesis, which results from competition for available Se and for components of the selenoprotein synthetic machinery [7,8], the levels of GPX1 and TR1 were also measured alongside those of GPX4. No significant differences in GPX1 activity were observed between cells of either genotype in either Se adequate or Se deficient cells after 5 days, similar to results with GPX4 (Fig. 4B). After 5 days of Se deficiency, GPX1 activity had decreased to around 47% (*p* < 0.05) of the activity in replete cells. GPX1 activity was further reduced (to 25% of the original level at d0) upon Se deficiency for additional 2 days (d7), which was significant for the CC genotype (*p* < 0.05) compared with d5 (Fig. 4B). In addition, when Se was added back to cells made Se deficient for 5 days, GPX1 activity was increased by 49% in cells with the TT genotype but not in cells with the CC genotype after 2 days (*p* < 0.05; Fig. 4B). After 4 days of Se repletion (d9), the GPX1 activity of the TT genotype cells was 90% greater than the GPX1 activity in cells of the CC genotype (*p* < 0.01; Fig. 4B).

TR1 protein levels were found to be higher (65%) in cells with the CC genotype compared to cells with the TT genotype under Se-adequate conditions (*p* < 0.05; Fig. 4C). However, Se deficiency for 5, 7, or 9 days did not result in any significant differences in TR1 levels between genotypes (Fig. 4C). After Se repletion, TR1 levels in cells with the TT genotype increased (by 63%; *p* < 0.05), whereas TR1 in cells with the CC genotype showed no increase and remained low (Fig. 4C).

### 3.5 Effect of GPX4c718t SNP in HUVEC on cellular lipid peroxidation levels

**Figure 5:**
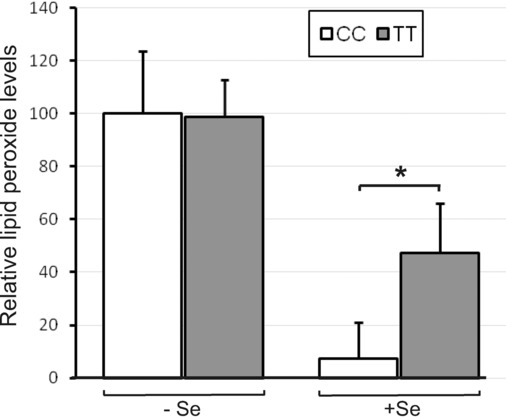
Effect of GPX4 718 SNP on lipid peroxide levels. HUVECs genotyped for GPX4c718t (CC or TT) were grown to confluence under Se deficient or replete conditions (−Se and +Se) and levels of lipid peroxides were measured using C11-BODIPY^581/591^. Lipid peroxide levels are expressed as a percentage of those in cells of the CC genotype without Se. Values shown are means ± SEM. Student's *t*-test was used to test for significant differences (*p < 0.05).

Since HUVECs containing either TT or CC at position 718 of the GPX4 gene demonstrated differences in antioxidant selenoprotein expression, levels of oxidative stress were measured in cells with respect to the GPX4c718t SNP using C11-BODIPY^581/591^. This latter reagent is a lipophilic reporter probe, which is incorporated into membranes where it is mainly oxidized by chain-propagating species such as peroxyl and alkoxyl radicals [22]. When cells were grown in the absence of Se, there were no differences in the level of lipid peroxides in cells of either genotype. However, in the presence of Se, cells with the CC genotype had much lower (15%) levels (*p* < 0.05) of lipid peroxides compared to cells with the TT genotype (Fig. 5). Overall, lipid peroxide levels in CC cells were lower in the presence of Se (*p* < 0.01) compared to levels in the absence of Se, whereas in TT cells, the presence of Se had no significant effect (Fig. 5). When cells were challenged with TBH, levels of lipid peroxides were significantly increased but there was no effect of the GPX4c718t SNP (not shown).

### 3.6 Effect of GPX4c718t SNP in HUVEC on cell viability

**Figure 6:**
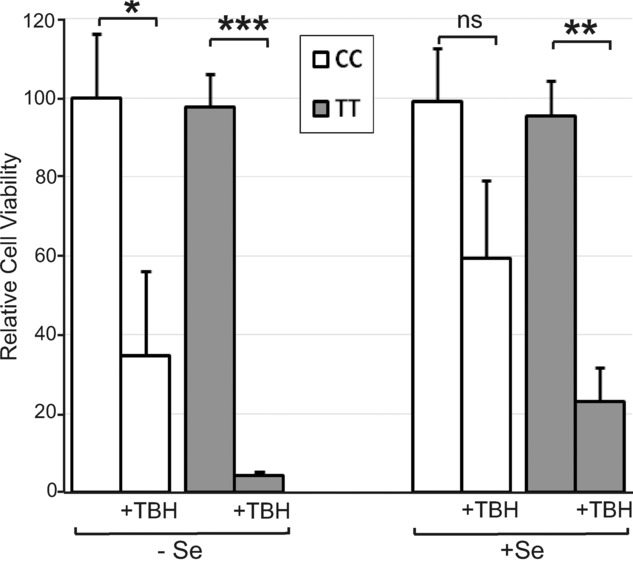
Effect of the GPX4 718 SNP and Se availability on cell viability. Genotyped HUVECs (CC or TT at GPX4 718) were grown for 4 days in M199 medium with 10% FCS in the presence or absence of 40 nM Se. Cell viability was measured after 4 h treatment with 0.3 mM TBH and is shown as a percentage of untreated CC cells grown in the absence of Se. Values shown are means ± SEM. Student's *t*-test was used to test for significant differences (*p < 0.05, **p < 0.01, ***p < 0.001).

Since selenoprotein expression appeared different in HUVECs of the CC and TT genotype depending on the Se supply, experiments were carried out to assess if the GPX4c718t genotype affected cell viability and if this was modified by Se availability. HUVECs of either genotype showed no change in cell viability in the presence or absence of Se (Fig. 6), therefore viability after an oxidative challenge was also measured. HUVEC viability was reduced in cells of either genotype after challenge with the oxidant TBH in the absence of Se (Fig. 6). In the presence of Se, the viability of cells with the CC genotype was not significantly affected after TBH challenge but the viability of cells with the TT genotype was reduced by 75% (*p* < 0.01; Fig. 6).

## 4 Discussion

Earlier studies have provided evidence that the T/C variation rs713041 within the *GPX4* gene region corresponding to the 3′-UTR of the mRNA has functional consequences [9,11,13,14]; the variants have distinct protein binding properties and homozygous carriers show altered selenoprotein levels and different levels of 5-lipoxygenase total products within lymphocytes. The present results support and extend these observations by showing that the SNP is functionally active in relation to adhesion events in vascular cells, which may have relevance to cardiovascular health and disease progression.

In the presence of Se, EC homozygous for the T-variant of the GPX4c718t SNP were shown to be more sensitive to oxidative challenge and to have higher levels of lipid hydroperoxides compared with cells homozygous for the C-variant. Conversely, there was no effect of the SNP on the sensitivity to oxidative challenge or lipid peroxide levels in the absence of added Se. Both EC and monocytes from volunteers homozygous for the T-variant of the GPX4c718t SNP were also shown to exhibit increased levels of adhesion compared to cells homozygous for the C-variant. Moreover, the effects of the T-variant of the GPX4c718t SNP on monocyte-EC adhesion in vivo will likely be additive to the separate effects measured here using nonhomologous cell types with respect to the GPX4c718t SNP. Monocytes are the primary inflammatory cells localized to human atherosclerotic plaques and monocyte binding to EC is an important step in atherosclerosis development [20,23]. Consequently, the finding that EC and monocytes from individuals with the TT genotype show both increased sensitivity to oxidative challenge and increased adhesion suggests that these individuals may have a greater susceptibility to endothelial damage and activation and therefore increased potential risk of associated disease.

TR1 has been suggested to be particularly important in protecting EC from *tert*-butyl hydroperoxide induced oxidative damage due to its relative abundance and ability to detoxify some damaging lipid hydroperoxides more efficiently than GPXs [24,25]. Interestingly, our findings support such a role for this selenoprotein since EC homozygous for the C-variant of the SNP expressed higher amounts of TR1 but not GPX1 or GPX4 under Se-adequate conditions (Fig. 4C). Additionally, increased levels of plasma TR1 protein have been observed previously in female volunteers with the CC genotype after supplementation with Se for 6-wks compared to females with the TT genotype [9].

The GPX4c718t SNP was also found to differentially affect GPX1 activity in EC under Se deficiency and repletion and overall there was also a trend for cells with the TT genotype to respond better to Se repletion by increasing TR1 and GPX4 protein levels. The finding that a SNP in GPX4 affects the expression of other genes such as TR1 and GPX1 can be explained by the hierarchical nature of selenoprotein expression in response to Se supply. Selenoproteins can compete between themselves for available Se (selenocysteine) and for components of the selenoprotein synthetic machinery [6–8]. This competition is driven, at least in part, by differences in the sequence of the selenoprotein 3′-UTRs and therefore potential polymorphisms within these regions have the ability to change the hierarchal order and alter expression of other selenoproteins. Since earlier work has shown the C-variant to be more effective than the T-variant in incorporating Se within a reporter construct [13], we hypothesise that upon Se repletion, GPX1 activity is higher in EC expressing the T-variant since the C-variant is better able to compete against GPX1 for incorporation of Se into GPX4. In this regard, it is important to note that human volunteers who are TT or CC for this variant in GPX4 show different responses in the GPX1/GPX4 ratio following changes in Se supplementation and RNA–protein binding assays in vitro indicate that the C-variant competes more strongly for binding proteins than the T-variant or the GPX1 3′-UTR [9]. Preliminary screening of human gene expression microarrays comparing EC cells of the CC versus the TT genotype also identified three other selenoproteins that were differentially expressed with the GPX4c718t SNP (GPX3, SELW1, and SELV) demonstrating further evidence for effects of the SNP on the selenoprotein hierarchy (data not shown).

Monocyte attachment to EC occurs through the expression of adhesion molecules (CAM) upon the EC surface in response to multiple stimuli including TNF-α [20]. Measurement of the levels of VCAM-1 protein (at least after ARA pretreatment) showed a strong correlation between the reduction in TNF-α-induced monocyte adhesion and lower expression of VCAM-1 in HUVEC with the CC genotype compared to cells with the TT genotype, indicating a likely mechanism for the genotype effect. CAM expression involves the activation of the NF-κB transcription factor [26] and previous studies have shown that Se can attenuate binding between these cell types [27] and inhibit NF-κB signaling [28,29]. Measurement of NF-κB activation in cells with respect to the SNP would shed further light on the mechanism involved. Overexpression and knock-down studies have implicated GPXs in the inhibition of NF-κB [28–30], but the precise role of Se and of the individual selenoproteins in this process have not been defined. As we have shown that the GPX4c718t SNP can alter the selenoprotein hierarchy it is likely that the observed effects on monocyte adhesion are mediated through changes in selenoprotein expression and subsequent differential attenuation of NF-κB activation.

The GPX4c718t SNP was also shown to interact with fatty acid treatment and to modulate the effect of certain PUFA on monocyte adhesion to EC. Upon a pro-inflammatory challenge, the PUFA-induced reduction in monocyte adhesion to EC was impaired under Se-deficient conditions in EC homozygous for the T-variant (Fig. 1). This was also apparent for binding of monocytes homozygous for the T-variant to EC treated with ARA (Fig. 3). Dietary fatty acids have been shown to modulate monocyte adhesion to EC both in vitro [17,18,31] and in vivo [32] and they have also been shown to modulate Se status through regulation of selenoprotein expression [19]. These results indicate that the beneficial effects of certain dietary fatty acids in attenuating interaction of monocytes with the EC may be reduced in individuals with the TT genotype, particularly when dietary Se is limiting. This finding provides further evidence of a likely adverse effect on vascular health that individuals with the TT genotype exhibit compared with individuals with the CC genotype.

In conclusion, the results presented here strengthen the evidence for the GPX4c718t SNP being functional and indicate that variants of this SNP can have detrimental consequences on endothelial function. In particular, EC homozygous for the T-variant of the SNP are more sensitive to oxidative challenge and show elevated levels of monocyte adhesion in response to dietary components compared to individuals homozygous for the C-variant of the SNP. Together these findings suggest that individuals with the TT genotype have inferior EC function and are therefore potentially more prone to disease. Additional studies are needed to define how the SNP affects the overall selenoprotein hierarchy and modifies individual selenoprotein function in the vasculature in order to fully characterize its impact on cardiovascular health and disease susceptibility.

## Abbreviations

ARA: arachidonic acid
CVD: cardiovascular disease
DHA: docosahexaenoic acid
EC: endothelial cell
FCS: fetal calf serum
GPX4: glutathione peroxidase 4
HUVEC: Human umbilical vein endothelial cell
LA: linoleic acid
3′-UTR: 3′-untranslated region
Se: selenium
Sec: selenocysteine
SNP: single nucleotide polymorphism
TBH: *tert*-butylhydroperoxide
TR: thioredoxin reductases
